# Supplementary material for: Can Menzerath’s law be a criterion of complexity in communication?
Source: PLoS One. 2021 Aug 20;16(8):e0256133. doi: 10.1371/journal.pone.0256133 (PMC8378695; doi:10.1371/journal.pone.0256133)
Supplement: S1 Fig — Gray solid lines indicate the MAL for each book analyzed separately, and the average MAL with black big circles and the solid line is just included for visual representation. In contrast, blue square points represent MAL computed directly for the full corpus treated as a single text. For all cases results are almost the same and there are hardly any differences between both methods. (PDF) [file pone.0256133.s001.pdf]

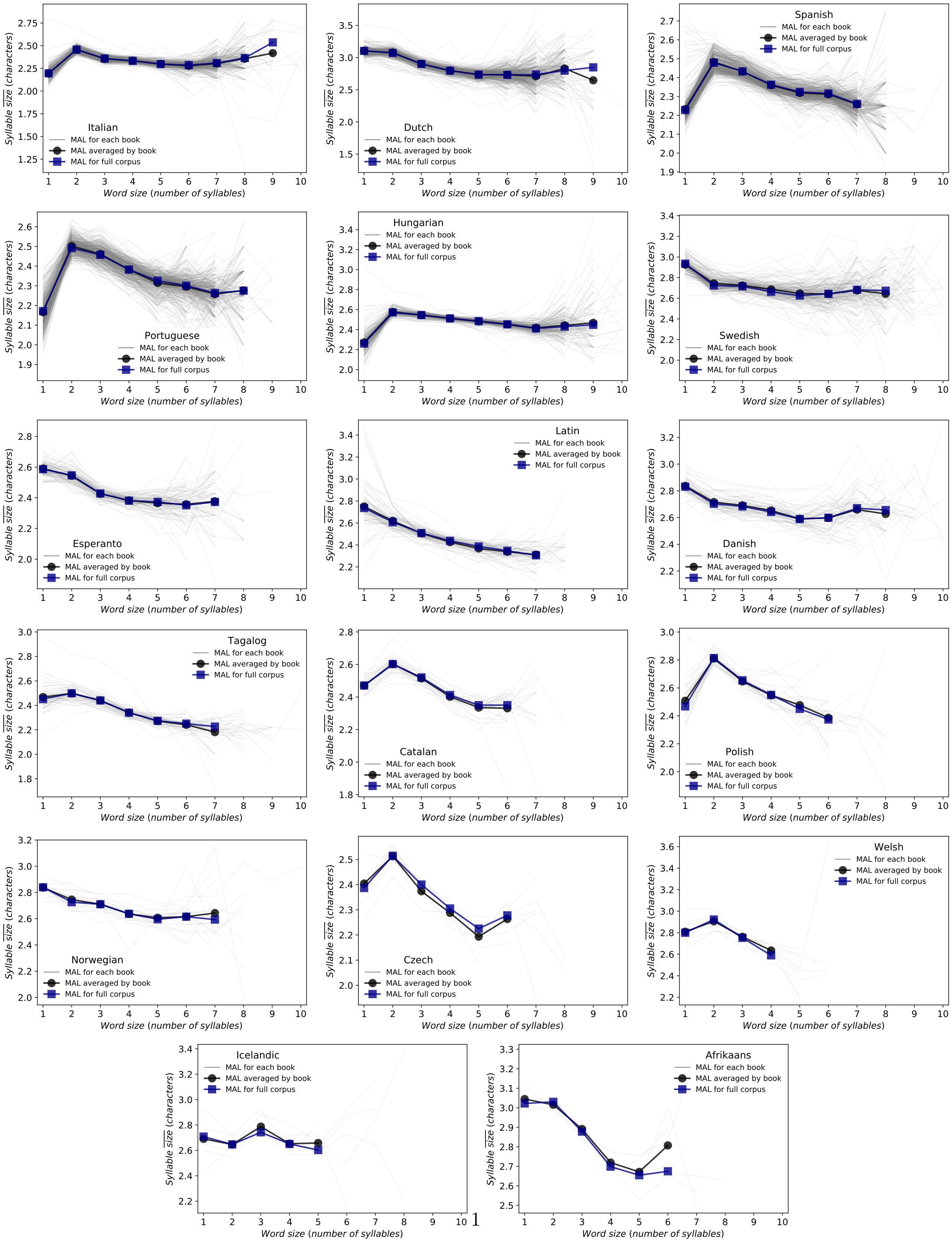

S1 Fig. Menzerath-Almann's law for full corpus vs averaging by book for all studied languages from the standardized Gutenberg project, omitting those presented in the main text. Gray solid lines indicate the MAL for each book analyzed separately, and the average MAL with black big circles and the solid line is just included for visual representation. In contrast, blue square points represent MAL computed directly for the full corpus treated as a single text. For all cases results are almost the same and there are hardly any differences between both methods.
