# Supplementary material for: Can Menzerath’s law be a criterion of complexity in communication?
Source: PLoS One. 2021 Aug 20;16(8):e0256133. doi: 10.1371/journal.pone.0256133 (PMC8378695; doi:10.1371/journal.pone.0256133)
Supplement: S2 Fig — It has been usual in the literature to exclude in the study of MAL elements with only one constituent (in this case words with only one syllable) as it is known that sometimes they do not follow Menzerath’s law. However, we here include experimental data and fits for Menzerath-Altmann’s law in the standardized Gutenberg corpus for languages not included in the main text, omitting the first point (monosyllables) and comparing with the memoryless source baseline. (PDF) [file pone.0256133.s002.pdf]

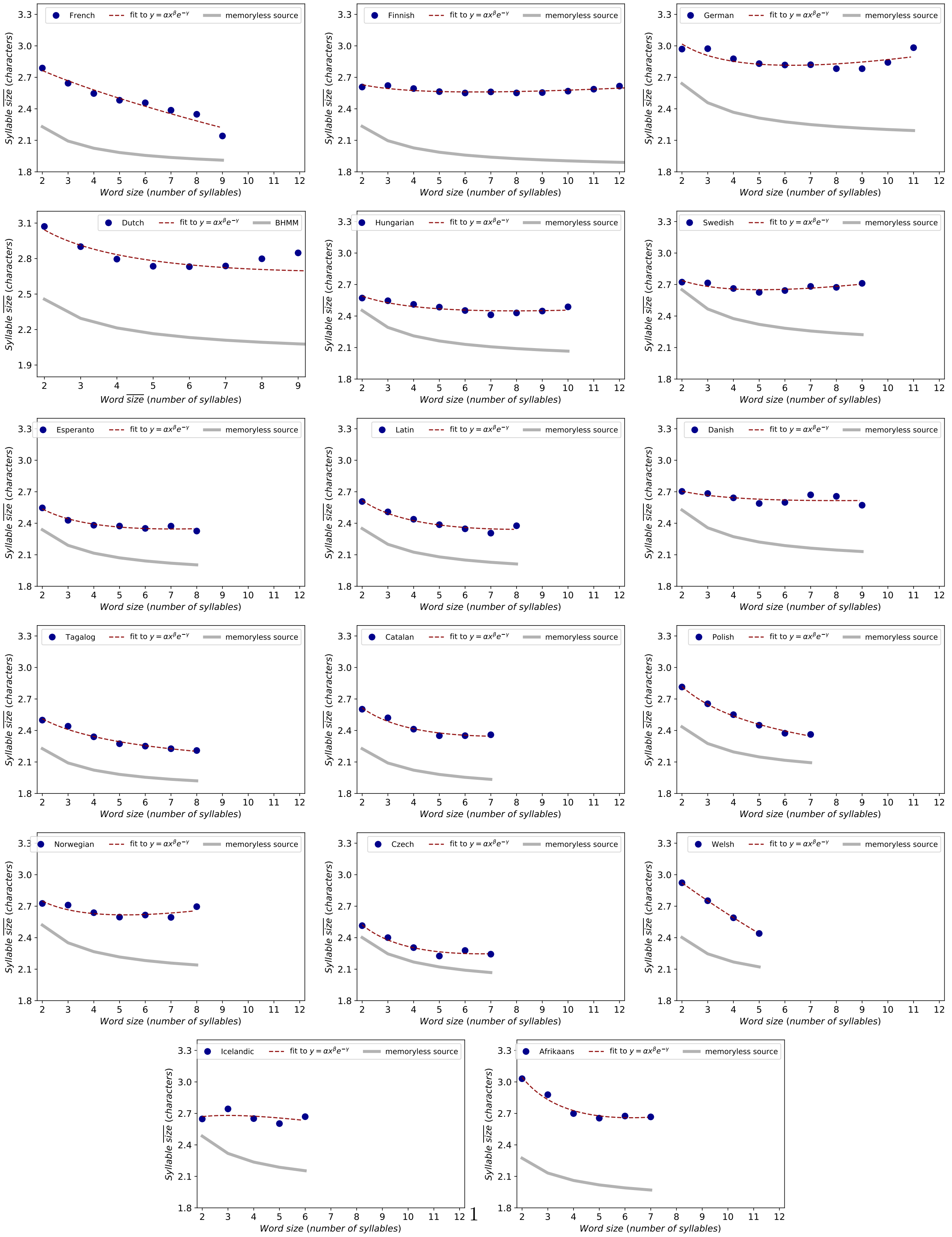

S2 Fig. Menzerath-Altmann's law and memoryless source baseline for full corpus excluding monosyllables. It has been usual in the literature to exclude in the study of MAL elements with only one constituent (in this case words with only one syllable) as it is known that sometimes they do not follow Menzerath's law. However, we here include experimental data and fits for Menzerath-Altmann's law in the standardized Gutenberg corpus for languages not included in the main text, omitting the first point (monosyllables) and comparing with the memoryless source baseline.
