# Supplementary material for: Can Menzerath’s law be a criterion of complexity in communication?
Source: PLoS One. 2021 Aug 20;16(8):e0256133. doi: 10.1371/journal.pone.0256133 (PMC8378695; doi:10.1371/journal.pone.0256133)
Supplement: S3 Fig — It can be seen that, depending on the language, Menzerath’s law is satisfied and the memoryless source model is a good baseline. (PDF) [file pone.0256133.s003.pdf]

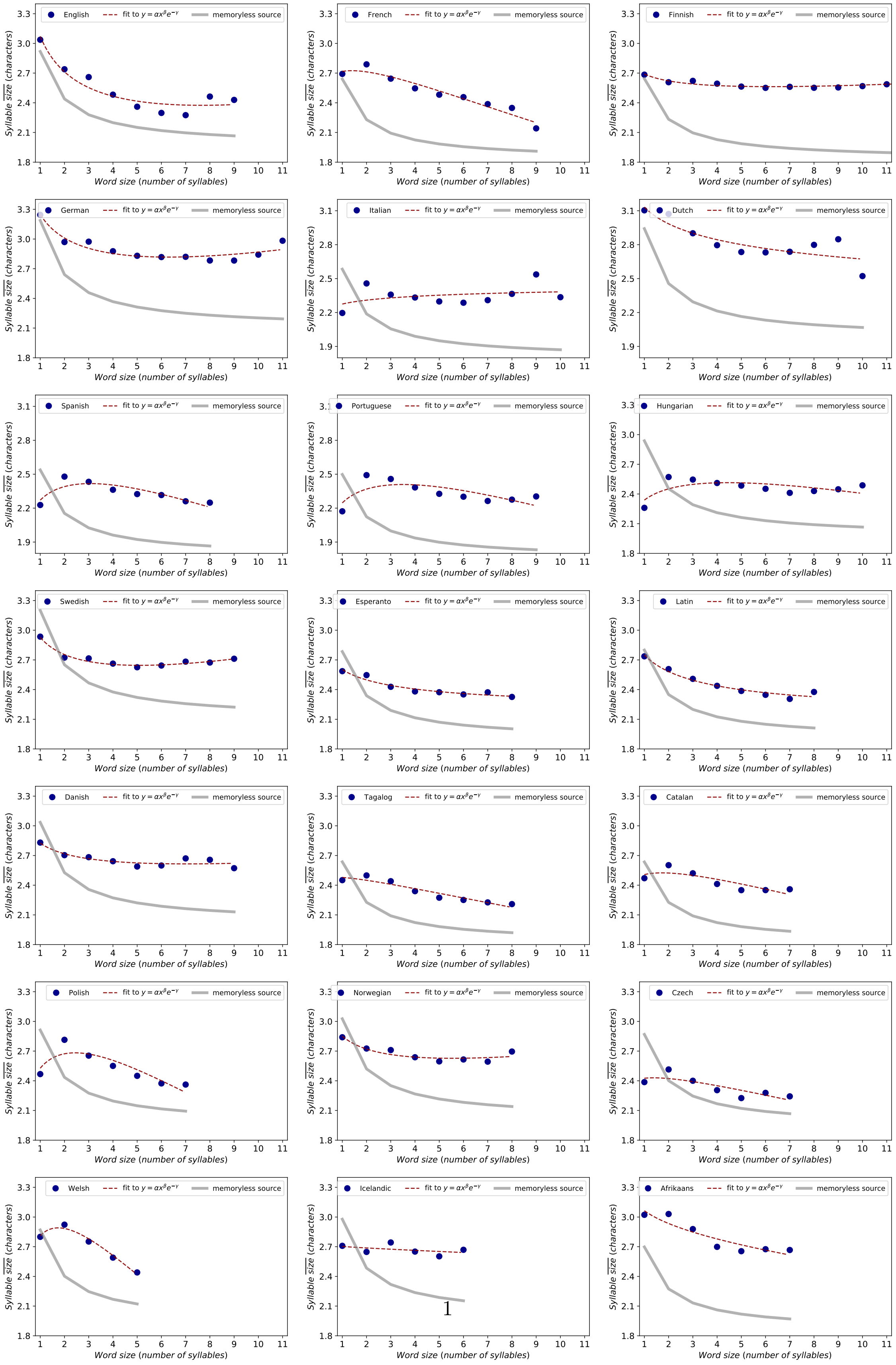

S3 Fig. Menzerath-Altmann's law and memoryless source baseline for full corpus including monosyllables. It can be seen that, depending on the language, Menzerath's law is satisfied and the memoryless source model is a good baseline.
