# Supplementary material for: Can Menzerath’s law be a criterion of complexity in communication?
Source: PLoS One. 2021 Aug 20;16(8):e0256133. doi: 10.1371/journal.pone.0256133 (PMC8378695; doi:10.1371/journal.pone.0256133)
Supplement: S4 Fig — The size of the word has been normalized where 0 corresponds to the first syllable and 1 corresponds to the last syllable. The mean size of monosyllabic words is represented with a black circle in x = 0.5. (PDF) [file pone.0256133.s004.pdf]

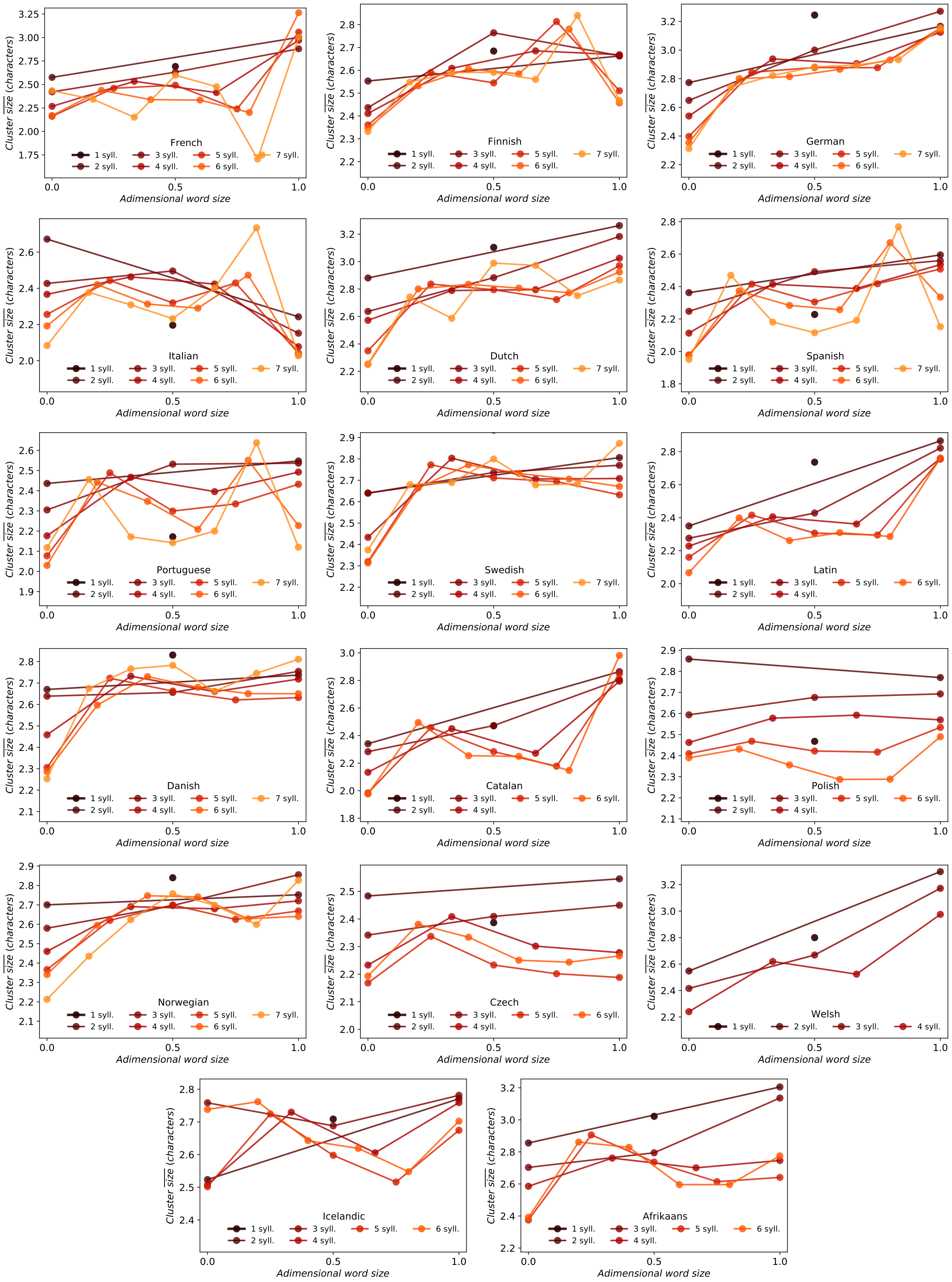

S4 Fig. Syllable sizes depending on word length and position in the word for 17 languages not shown in the main text. The size of the word has been normalized where 0 corresponds to the first syllable and 1 corresponds to the last syllable. The mean size of monosyllabic words is represented with a black circle in $x = 0.5$.
